# Dermoscopic and Reflectance Confocal Microscopy Features of Superficial Morphea on Preexisting Atrophoderma of Pasini and Pierini

**DOI:** 10.5826/dpc.1202a48

**Published:** 2022-04-01

**Authors:** Chun-Xiao Song, Yu-Ting Zhang, Cheng Tan

**Affiliations:** 1 Department of Dermatology, Affiliated Hospital of Nanjing University of Chinese Medicine, Nanjing, China

**Keywords:** morphea, atrophoderma, hyperpigmentation, hypopigmentation, dermatology

## Introduction

Superficial morphea (SM) is a distinct new variant of localized scleroderma with the collagen proliferated and deposited in the papillary and upper reticular dermis [1]. We present a case of SM developed on a primary lesion of atrophoderma of Pasini and Pierini (APP), and describe related dermoscopic and reflectance confocal microscopy (RCM) features.

## Case Presentation

**Figure 1:**
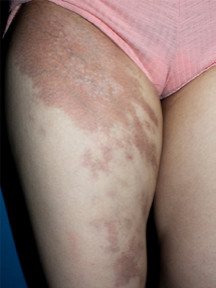
Large hyperpigmented patches were distributed in a zosteriform pattern on the right groin area, and the thigh. Lesions were atrophic and slightly depressed without induration. Multiple 2 to 3 mm depigmented macules speckled within the hyperpigmented patch are shown.

**Figure 2:**
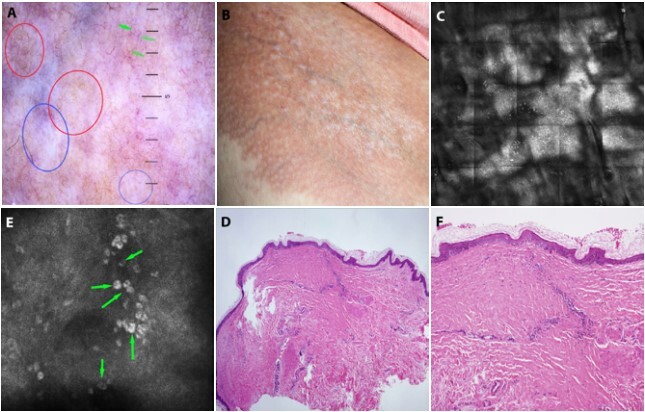
Dermoscopic examination of the white spots showed whitish fibrotic beams and linear arborizing vessels (green arrows). (A, B) The pigment network is irregularly distributed with a storiform pattern (red oval circles) in some speckled hypopigmented macule (blue oval circles). (C) RCM mosaic shows marked hyperreflective areas with decreased appendageal structures. Histological examination of this hypopigmented macule displayed decreased epidermal thickness with flattened rete pegs. (D) mild superficial perivascular lymphocytic infiltrate and pronounce dense clumping and homogenization of collagen bundles, compared to the neighboring area’s unaltered collagen. (E, F) Discrete, highly reflective clouds in the “coffee-bean” pattern under RCM, which might be corresponding to the clumps of collagen under the microscope.

A 26-year-old Chinese woman presented with a seven-year-history of large hyperpigmented patches on the right leg. On examination, larger hyperpigmented patches were distributed in a zosteriform pattern. It spread from the right groin area, down the medial portions of the thigh, and finally to the popliteal fossa region. Furthermore, multiple 2 to 3 mm depigmented macules were disseminated within these hyperpigmented patches (Figure 1). Dermoscopic examination of the white spots showed whitish fibrotic beams and linear arborizing vessels. The pigment network was irregularly distributed with a storiform pattern in some speckled hypopigmented macules. Histological examination of a hypopigmented macule displayed decreased epidermal thickness with flattened rete pegs. There were mild superficial perivascular lymphocytic infiltrate and pronounce dense clumping and homogenization of collagen bundles, compared to the neighboring area’s unaltered collagen. Discrete, highly-reflective clouds were in the “coffee-bean” pattern under RCM, which might be corresponding to the clumps of collagen in histopathology (Figure 2). These findings were consistent with the diagnosis of SM (over APP), and the application of 1% pimecrolimus ointment for 4 months showed no improvement.

## Discussion

SM is typically presented with symmetric hypopigmented to hyperpigmented patches at intertriginous sites. Histologically, there are flattened rete ridges. The collagen fibers in the upper reticular dermis become thickened or homogenized, with the deeper dermis’s invariable sparing. Perivascular lymphocytic infiltration is present in the superficial dermis with occasional plasma cells [2]. Dermoscopic examination of the white spots in our patient showed whitish fibrotic beams and linear arborizing vessels. The pigment network is irregularly distributed with a storiform pattern in some speckled hypopigmented macules. RCM mosaic shows marked hyperreflective areas with decreased appendageal structures.

Divergent opinions about the relationship between SM and APP are present in literature. Some authors believe that SM is not identical to APP considering the clinical depression of “cliff sign” and older age of onset in APP. SM differs itself from APP with thickened collagen in upper reticular dermis. Others believe SM and APP belong to the same entity mainly considering both share a chronic benign course and a favorable prognosis that usually needs no treatment [3,4]. Besides, APP and SM may coexist as separate entities in the same patient or association with classical morphea or systemic scleroderma, and therefore APP can be considered an abortive type of scleroderma without sclerosis [4].

## Conclusions

The presence of discrete hypopigmented macules of SM within the primary lesion of APP adds another evidence that SM and APP are part of the same spectrum of disease. The marked hyperreflective areas with discrete, highly reflective clumped as white coffee beans. Dermoscopy and RCM can be applied as an ancillary diagnostic technique in SM.
